# Do consumer‐mediated negative effects on plant establishment outweigh the positive effects of a nurse plant?

**DOI:** 10.1002/ece3.3935

**Published:** 2018-03-06

**Authors:** Tomohiro Fujita, Chisato Yamashina

**Affiliations:** ^1^ Center for Environmental Biology and Ecosystem Studies National Institute for Environmental Studies Tsukuba Japan; ^2^ Faculty of Life and Environmental Sciences University of Tsukuba Tsukuba Japan

**Keywords:** apparent competition, facilitation, Forest–savanna boundary, miombo woodland, nurse plant, seed predation

## Abstract

Many studies demonstrated the importance of facilitative effect by nurse plant on seedling establishment. Few studies evaluated the negative effects of consumers on plant establishment under nurse plants by dealing with them during multiple demographic processes. We investigated the balance between the facilitative effect and negative effects of consumers during multiple demographic processes in Malawi in southeastern Africa. We chose *Ficus natalensis* as a nurse plant and compared it with three other microsites in tropical woodlands: *Brachystegia floribunda* (a dominant woodland species), *Uapaca kirkiana* (a woodland species), and a treeless site. We quantified the seed rain, postdispersal seed predation, germination, and seedling survival of *Syzygium guineense* ssp. *afromontanum* (a common forest species). Within each microsite, we quantified the overall probability of recruitment. We also measured seedling abundance of *S. guineense* ssp. *afromontanum*. We found that *Ficus natalensis* exerted both positive and negative impacts on the establishment of *S. guineense* ssp. *afromontanum*. *Ficus natalensis* facilitated seed deposition, seed germination, and seedling survival. On the other hand, seed removal at postdispersal stage was highest under *F. natalensis*. Interestingly, *B. floribunda* also had positive effects on germination and seedling survival, but not on seed deposition. When we excluded the seed arrival stage from our estimation of the recruitment probability, the highest value was found under *B. floribunda*, not under *F. natalensis*. When we included the seed arrival stage, however, the order of recruitment probability between *F. natalensis* and *B. floribunda* was reversed. The probability was one order of magnitude higher under *F. natalensis* than under *B. floribunda*. Our estimation of the probability which included the seed arrival stage was consistent with natural patterns of *S. guineense* ssp. *afromontanum* establishment. Despite the presence of opposite effects, the net effects of *F. natalensis* on *S. guineense* ssp. *afromontanum* recruitment in tropical woodlands can be positive.

## 1 INTRODUCTION

Facilitation by nurse plants is now widely recognized as an important mechanism in the structuring of plant communities (Bruno, Stachowicz, & Bertness, 2003). Direct facilitation among plants takes place when a certain plant species ameliorates microclimate conditions or increases resource availability for another plant species beneath its crown (Flores & Jurado, 2003). Likewise, indirect facilitation occurs when other organisms, such as pollinators and seed dispersers, mediate interactions between nurse plants and the beneficiary species (Carlo & Aukema, 2005; Ghazoul, 2006). However, evidence shows that competition by nurse plants may offset the positive effects, depending on the life history stage of both the nurse plant and the beneficiary species (Ibez & Schupp, 2001; Cavieres & Penaloza, 2012). In addition, consumer‐mediated indirect effects may disrupt facilitation when the nurse plant attracts consumers by offering shelter or food, such as seeds and seedlings (Rolhauser, Chaneton, & Batista, 2011).

Indirect negative interactions mediated by the action of consumers represent so‐called apparent competition (Holt, 1977). Recently, several studies have suggested that apparent competition can be crucial in determining plant recruitment (Rand, 2003) and invasion by exotic plant species (Orrock, Witter, & Reichman, 2008). However, few studies have applied the concept of consumer‐mediated negative effects when examining plant recruitment, where facilitation is thought to prevail (Chaneton, Noemi Mazía, & Kitzberger, 2010; Rolhauser et al., 2011). Chaneton et al. (2010) demonstrated that while shrub cover decreased the desiccation mortality of *Austrocedrus chilensis* seedlings, it also increased beetle‐mediated damage to the seedlings. Rolhauser et al. (2011) found that palm trees promoted the deposition of bird‐dispersed *Allophylus edulis*, while the seedlings beneath the palm tree were exposed to a greater risk of mortality due to physical damage by wild animals, such as wild boars and armadillos, which are presumably attracted by palm seeds and/or seedlings. Although these previous studies shed light on the negative effects of consumers under nurse plants, few studies have simultaneously considered all initial stages of recruitment, from seed arrival to seedling survival (see Smit, den Ouden, & Díaz, 2008).

*Ficus* trees (Moraceae) in tropical savannas, woodlands, and postagricultural fields are widely known to play a role as nurse plants for tropical forest tree species (Fujita, 2014, 2016; Schlawin & Zahawi, 2008; Slocum, 2001). In some cases, *Ficus* trees drive a “nucleation” process, whereby forest species tend to clump around the *Ficus* trees, leading to the formation of forest patches. Several studies have demonstrated the role of the direct and indirect facilitative effects of *Ficus* trees on the process of nucleation (Fujita, 2014, 2016; Schlawin & Zahawi, 2008; Slocum & Horvitz, 2000). However, few have considered consumer‐mediated negative effects, nor the net outcome of the positive and negative effects on plant establishment over multiple demographic stages.

We investigated the balance between facilitation and the negative effects of consumers during multiple demographic processes in a tropical woodland area in northern Malawi in southeastern Africa. In northern Malawi, circular forest patches can be found within tropical woodland and large individuals of fleshy fruit trees, especially *Ficus natalensis*, generally exists in the centres of these patches (Fujita, 2014); these forest patch structures are a common characteristic of “nucleated forest patches” rather than fragmented forests (Duarte, Carlucci, Hartz, & Pillar, 2007; Favier, De Namur, & Dubois, 2004). We quantified the seed rain, postdispersal seed predation, seed germination, and seedling survival of *Syzygium guineense* ssp. *afromontanum* (a common forest tree species) under *F. natalensis* and at three other microsites. We sought to answer two main questions: (1) During the multiple demographic processes, is there any evidence of negative effects from consumers on the establishment of *S*. *guineense* ssp. *afromontanum* under *F. natalensis* and (2) if so, what is the net outcome of the positive and negative effects on plant establishment?

## 2 MATERIAL AND METHODS

### 2.1 Study area

We conducted this study in northern Malawi in southeastern Africa. In southeastern Africa, 2.7 million km^2^ are covered with tropical woodlands called miombo woodland. Miombo woodland consists of leguminous species in three closely related genera: *Brachystegia*,* Julbernardia*, and *Isoberlinia* (Campbell, Frost, & Byron, 1996). Miombo woodland has 10–20 m tall canopy and generally consists of deciduous trees. The woodlands typically have continuous herbaceous layer of C4 grasses. In this region, montane rain forests can also be found on mountain crests and in valleys (White, Dowsett‐Lemaire, & Chapman, 2001). The forests consist of evergreen trees and have a tall canopy (20–25 m) with numerous lianas.

The study site was located in a rural zone managed by a local village (10°58′S, 34°04′E) on the north Viphya Plateau in northern Malawi. The mean annual rainfall is over 1,270 mm on the north Vipya Plateau (Chapman, 1970). Most rainfall occurs during the rainy season between December and April. The soil in the study site is a well‐drained red and sandy clay loam. Although miombo woodlands predominantly cover the study site, some montane rain forests exist on mountain crests (>1,800 m asl) and in valleys. In addition, several circular forest patches (~10–1,800 m^2^) consisting of montane rain forest tree species occur within miombo woodland (1,700–1,800 m asl; see Fujita, 2014 for details).

In this area, the local people burn most of the miombo woodland to clear footpaths approximately every 2–3 years. Generally, these fires are set between September and December (late dry season). Fire is not likely to enter far into the montane rain forest because of the dense canopy, which excludes grasses and maintains a more humid understory (Hoffmann et al., 2012). Few trees are cut from either miombo woodlands or montane rain forests because they are located far from settled areas.

### 2.2 Focal forest tree species

We used *Syzygium guineense* ssp. *afromontanum* (Myrtaceae) in our experimental and observational studies. *S*. *guineense* ssp. *afromontanum* is endemic to montane rainforests and is common on the Vipya Plateau (White et al., 2001). In this study site, *S*. *guineense* ssp. *afromontanum* also commonly occur in forest patches within miombo woodland. *S*. *guineense* ssp. *afromontanum* is a medium to tall evergreen tree up to 30 m in height. This species produces purple berries during the rainy season (from January to March in the study area). The fruit and seed sizes are 1.6 × 1.4 cm and 1.2 × 1.1 cm, respectively (*n* = 6).

### 2.3 Characteristics of the studied microsites

We selected four microsites in the miombo woodlands: under *F. natalensis*, under *Brachystegia floribunda*, under *Uapaca kirkiana*, and in treeless sites. *F. natalensis* is a deciduous tree up to 20 m in height that occurs in eastern and southern Africa. *F. natalensis* occurs primarily in miombo woodlands but is also found in the center of circular forest patches in this area. Its syconia (1.1 × 1.0 cm, *n* = 10) turn yellow‐red when ripe. *F. natalensis* has two periods of ripening: August to October and January to April. The syconia are eaten by birds and mammals such as monkeys and fruit bats (Foard, Van Aarde, & Ferreira, 1994). *B. floribunda* (Fabaceae, Caesalpinioideae) is a medium size to tall deciduous tree up to 20 m in height. *B. floribunda* is the dominant tree species in the miombo woodlands of northern Malawi. *B. floribunda* produces pods from October to January, and the pods disseminate their seeds explosively. *U. kirkiana* (Phyllanthaceae) is a small to medium size tree (up to 13 m) that also commonly occurs in this study area. It grows fleshy fruit (2.6 × 2.6 cm, *n* = 7) with three or four ripe seeds from September to December in the study area. The treeless sites were areas in which there were no trees with crowns exceeding a 3 m radius or diameter at breast height (dbh) values >5 cm.

We selected eight *F. natalensis* individuals and chose *B. floribunda*,* U. kirkiana*, and the treeless sites within 50 m of the *F. natalensis*. We chose *B. floribunda* and *U. kirkiana* individuals with heights and dbh similar to those of the *F. natalensis*. *F. natalensis*,* B. floribunda*, and *U. kirkiana* exhibited little to no canopy overlap with other individuals. The mean distance between each microsite and the forest or forest patches was 169 m for *F. natalensis* (range, 56–307 m), 165 m for *B. floribunda* (range, 64–297 m), 170 m for *U. kirkiana* (range, 60–289 m), and 173 m for the treeless site (range, 64–332 m).

### 2.4 Data collection

#### 2.4.1 Seed deposition of *Syzygium guineense* ssp. *afromontanum*

From January to March 2012, we monitored seed rain of *S*. *guineense* ssp. *afromontanum* at four microsites using seed traps (70 × 70 cm). The traps were composed of fine‐mesh net and were fixed to the ground. Each seed trap had 5‐cm‐high sides to prevent the deposited seeds from washing away. We set three seed traps at each of four microsites (96 seed traps in total). Three seed traps were installed at 1 m from each trunk or at 1 m from the center of the treeless sites. The direction of the first quadrat was decided randomly, and the others were set at 120° and 240° from the first quadrat. Each microsite was checked twice a week to count *S*. *guineense* ssp. *afromontanum* seeds.

#### 2.4.2 Seed removal of *Syzygium guineense* ssp. *afromontanum*

For our seed removal experiment, *S*. *guineense* ssp. *afromontanum* fruits were collected from seven individuals in montane rain forests. We removed the fruit pulp manually and then checked for viability using a floating test. Ten seeds were placed in each of the four microsites, and the total number of seeds was 320. The seeds were placed on 8 × 8 cm pieces of fine‐meshed, gray netting that was secured at the corners using small nails (Holl, 2002). The netting was stapled at the corners to create a 1‐cm side, which prevented seeds from being washed away but allowed for entry of seed predators. The seed removal experiments began in late January 2012, that is, in the middle of the rainy season when *S*. *guineense* ssp. *afromontanum* is naturally dispersed throughout the study site. Remaining, intact seeds were counted after 3, 6, 12, 24, and 32 days.

We analyzed canopy openness, as previous studies have shown that vegetation openness is an important factor in seed removal. Several studies have found that the probability of seed removal is highest in more closed microhabitats where seed consumers may have more protection against large predators (Pérez‐Ramos & Marañón, 2008; Smit et al., 2008). Four hemispherical canopy photographs were taken at each microsite using a fish‐eye lens (Raynox DCR‐CF; Yoshida Industry, Tokyo, Japan). We took the pictures at the middle point of the crown radius along each cardinal direction from the trunk. At the treeless site, we took them in each cardinal direction at 1 m from the center of the plot. All pictures were taken at a height of 1 m above the ground. These photographs were taken in February 2012 after the leaves were fully expanded. We calculated canopy openness using gap light analyser software (Frazer, Canham, & Lertzman, 1999). The data at each microsite are presented as the average of the four values.

#### 2.4.3 Germination of *Syzygium guineense* ssp. *afromontanum*

Germination was monitored to determine the postdispersal fate of *S*. *guineense* ssp. *afromontanum* at the four microsites. Seeds were collected and treated in the same manner as described above. We sowed 25 viable seeds at each of the four microsites (800 seeds in total) in January 2012. To prevent seed predation, the sampling stations were covered with 50 × 50‐cm cages made of wire mesh. We considered a seed to have germinated if the radicle had emerged. Each microsite was monitored once a week for 10 weeks.

#### 2.4.4 Seedling survival of *Syzygium guineense* ssp. *afromontanum*

In January 2012, we planted *S*. *guineense* ssp. *afromontanum* seeds in a nursery. The seedlings were transferred to the four microsites 4 weeks after seeding. All seedlings had their true leaves when transplanted. Sixteen seedlings were planted in 4 plant × 4 plant grids with the seedlings separated by 50 cm. In total, 512 seedlings were used in the experiment. We watered the seedlings at once after transplanting, but we did not apply any treatments after that. We checked the survival 1 week after transplanting, and we replaced seedlings that had died probably because of transplant shock. We then monitored seedling survival at approximately 1, 6, 7, 10, 19, and 31 months after transplanting. Seedling death was assigned to the most evident cause. Seedlings that became brown and dried out without any visible damage were classified as dying due to drought (Plate 1a). Seedlings that lost the aboveground part and had visual signs of fire damage were classified as dying due to fire (Plate 1b). Seedlings attacked by insects (apparently cutworm [Noctuidae]) were classified as dying from insect damage. These seedlings were cut smoothly near ground level and only a small section was left aboveground near the base (Plate 1c). Most of these seedlings bore filaments of insects. Trampling by ungulates caused seedling death by direct stepping.

**Plate 1**

#### 2.4.5 Seedling number of *Syzygium guineense* ssp. *afromontanum*

We counted the seedling number of *S*. *guineense* ssp. *afromontanum* in four field microsites in August (before the fire season) in 2011. We set four 2 × 5 m quadrats in the four cardinal directions around each tree trunk or around the center of the plot at the treeless sites. All of the *S*. *guineense* ssp. *afromontanum* seedlings (0.2–1 m high) were counted in each quadrat, and the data were combined for each plot.

### 2.5 Data analysis

All statistical analyses were performed with R software (ver. 2.14.0; R Development Core Team, http://www.r-project.org/). A general linear mixed model (GLMM) with Poisson distributions and log‐link function was used to analyze the seed rain using glmer function in the R lmer4 package. This analysis included fixed effects of microsite type and random effects of individual seed trap. The final percent, seed removal, germination, and seedling survival were also analyzed using a GLMM with a binomial distribution and logit‐link function. These analyses included fixed effects of microsite type and random effects of individual microsite. Significant differences among the microsites were tested with Tukey's multiple comparison tests. Multiple comparisons were performed using the GLHT function in the R multicomp package.

Transition probabilities were calculated for each of the four microsites as the mean number of individuals completing a stage divided by the number of individuals entering that stage. The cumulative probability of recruitment (CP) for each habitat was defined as the product of the individual transition probabilities. For a given microsite, the probability of seed arrival was defined as the ratio of mean seed deposition to the sum of mean seed deposition at all four microsites. In this study, we did not track seed fate after seed removal. Although most of the removed seeds were eventually predated, some can germinate and be recruited after removal (Brewer & Rejmanek, 1999; Klinger & Rejmanek, 2009). If we had used the percentage of seed removal from our experiment in the transition probabilities analysis without considering it, we could have overestimated the overestimate seed survival stage. Therefore, we adjusted the value of the seeds remaining the probability of seed survival after seed removal from previous studies. We extracted the probability of seed survival from several previous studies that monitored seed fate at the postdispersal stage in tropical regions and that examined plant species that had seeds similar in size to *S*. *guineense* ssp. *afromontanum* seeds (<30 mm in diameter) and had a fleshy pulp, such as berries and drupes. We extracted the values from several studies (Carvajal and Adler, 2008; Brewer and Rejmanek, 2009; Klinger and Rejmanek, 2009; Aliyu et al. 2014; Cao et al., 2016; Rosin and Poulsen, 2016). We multiplied our observed values (seed removal) by the mean value (48%) from these studies. Then, we added these adjusted seed survival values to the seeds remaining in experiments at all four microsites. We included these values as remained seed in the transition probability analysis (Figure 3).

## 3 RESULTS

### 3.1 Seed rain of *Syzygium guineense* ssp. *afromontanum*

**Figure 1:**
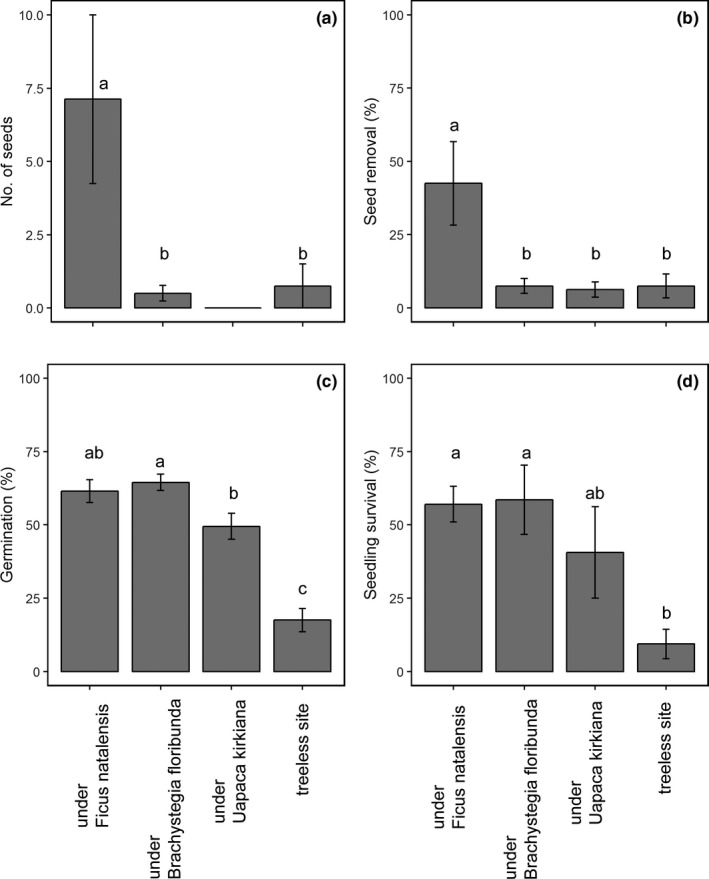
Mean number of dispersed seeds (a); removed seeds (b); mean seed germination (c); and mean seedling survival percentage (d) of *Syzygium guineense* ssp. *afromontanum* (±1 *SE*) at four microsites in northern Malawi. All microsites were located in miombo woodlands. Seed rain was monitored from January to March 2012. Seed removal (*n* = 320) was monitored from January to February 2012. Germination (*n* = 800) was monitored from January to March 2012. Seedling survival (*n* = 512) was measured from February 2012 to August 2014. Means with the same letter in superscript are not significantly different (*p* > .05) among the microsites based on Tukey's post hoc tests

No seed deposition of *S*. *guineense* ssp. *afromontanum* was found under the *U. kirkiana*; therefore, seed deposition data under *U. kirkiana* were excluded from the analysis. Of the *S*. *guineense* ssp. *afromontanum* seeds collected, 85% were recorded under *F. natalensis* (Figure 1a). There were significantly more seeds deposited under *F. natalensis* than under *B. floribunda* or in the treeless site. The number of seeds deposited under *F*. *natalensis* was 14 times higher than that deposited under *B*. *floribunda* and 10 times higher than at the treeless site.

### 3.2 Seed removal of *Syzygium guineense* ssp. *afromontanum*

**Figure 2:**
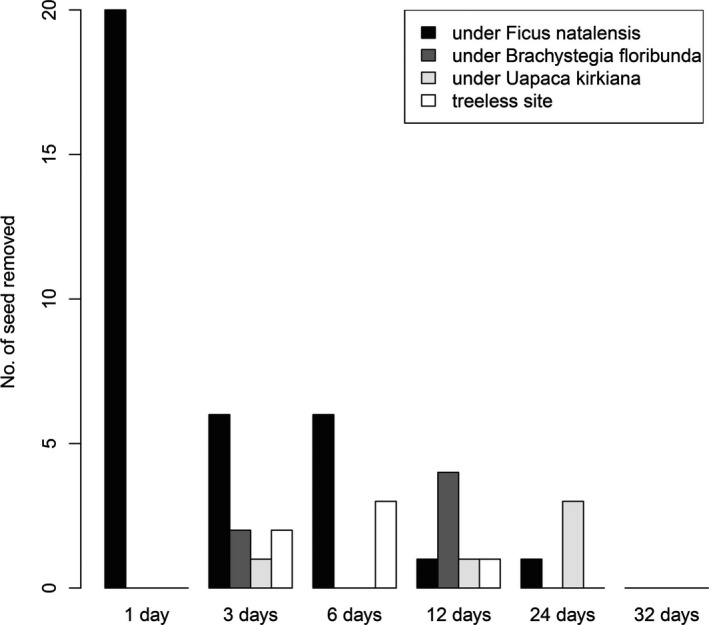
Seed removal rates of *Syzygium guineense* ssp. *afromontanum* 32 days after seed placement at four microsites in northern Malawi

Seed removal of *S*. *guineense* ssp. *afromontanum* differed among the microsites (Figure 1b). The percentage of seed removal under *F. natalensis* was significantly higher than at the other three microsites. Seed coat remnants likely eaten by rodents were often found around the experimental sets under *F. natalensis*, providing evidence of effective seed predation (Plate 2). All of the seeds were removed in two of the eight experimental sets at the *F. natalensis* microsites. Most of the seed removal occurred within 1 week after the seed setting (Figure 2).

**Plate 2**

Canopy openness was significantly lower under *F. natalensis* and *B. floribunda* compared to the other two microsites (Tukey's post hoc test, *p* < .05; data not shown). However, the values under *F. natalensis* were not significantly different from those under *B. floribunda* (Tukey's post hoc test, *p* = .43).

### 3.3 Germination of *Syzygium guineense* ssp. *afromontanum*

Overall, 48.3% of the seeds germinated until the end of the experiment, with higher percentages under *F. natalensis* and *B. floribunda* than at the treeless site (Figure 1c). The percentage of germination under *B. floribunda* was also significantly higher than that under *U. kirkiana*. Although that value under *F. natalensis* tends to be higher than that under *U. kirkiana*, there was no significant difference between them (Tukey's post hoc test, *p* = .12). No significant difference was found between *F. natalensis* and *B. floribunda* (Tukey post hoc test, *p* = .94).

### 3.4 Seedling survival of *Syzygium guineense* ssp. *afromontanum*

In total, 41.4% of seedlings survived after 31 months. The percentages of seedling survival under *F. natalensis* and under *B. floribunda* were significantly higher than those at the treeless site (Figure 1d). Again, no significant differences in seedling survival were recorded between *F. natalensis* and *B. floribunda* (Tukey's post hoc test, *p* = .99). The cause of seedling mortality was recognized for 252 seedlings (84% of the dead seedlings). Fire was the main cause of seedling mortality (43%), followed by drought (31%). Insect damage and trampling by ungulates caused just 9% and 1% of total mortality, respectively.

### 3.5 Overall probabilities of recruitment

**Figure 3:**
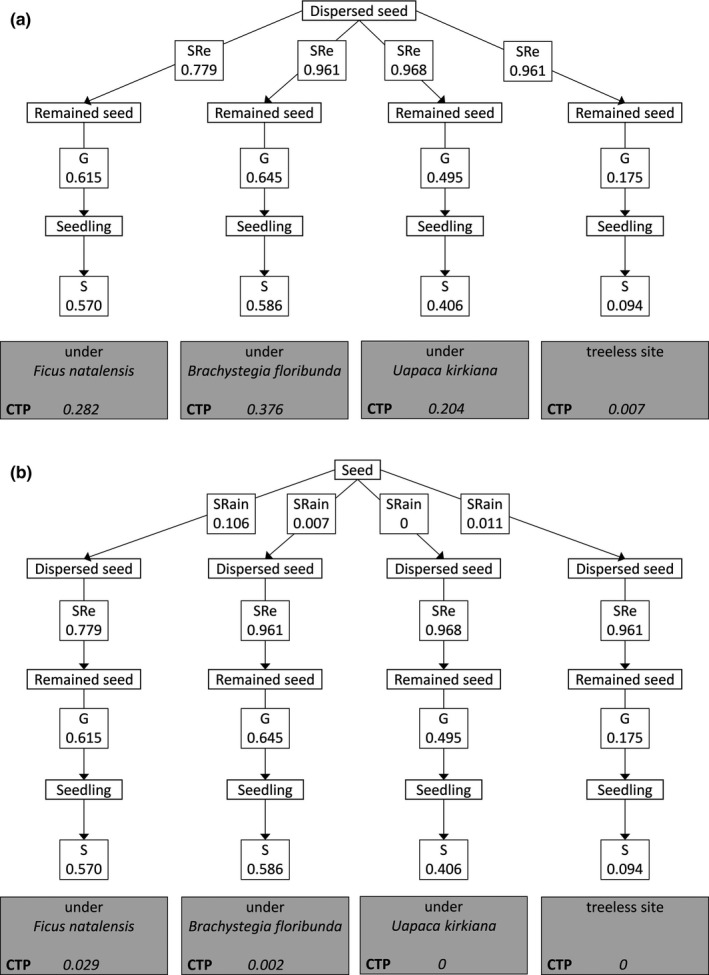
Transition probabilities between subsequent life stages of *Syzygium guineense* ssp. *afromontanum* at four microsites in northern Malawi. We excluded the seed arrival stages in Figure 3a in the estimation of the cumulative transition probabilities. In Figure 3b, we included the seed arrival stages. CTP, cumulative transition probability; SRain, seed rain; SRe, seed removal; G, germination; S, seedling survival

To examine the suitability of each microsite at the postdispersal stages, we first excluded the seed arrival stage from the estimation of transition probability (Figure 3a). The cumulative probabilities of recruitment were highest under *B. floribunda*, followed by *F. natalensis*,* U. kirkiana*, and at the treeless sites. The highest value under *B. floribunda* was attributed to higher germination and seedling survival and lower seed predation. When we included the seed arrival stage in the estimation of the transition probability, however, the order of the probability between *F. natalensis* and *B. floribunda* was reversed. The probability was one order of magnitude higher under *F. natalensis* than under *B. floribunda* (Figure 3b).

### 3.6 Natural distribution of *Syzygium guineense* ssp. *afromontanum* seedlings

No seedling of *S*. *guineense* ssp. *afromontanum* was recorded under *U. kirkiana* or at the treeless sites; therefore, for the seedling data, we excluded these microsites from the statistical analysis. The number of seedlings differed significantly between *F. natalensis* and *B. floribunda* (Wilcoxon test, *p* < .01). Most seedlings (95%) were found under *F. natalensis*.

## 4 DISCUSSION

Although some studies examined the negative effects of consumers on plant establishment under nurse plants (Chaneton et al., 2010; Rolhauser et al., 2011), these investigations focused on a single life stage, especially as concerns seedling survival (but see Smit et al., 2008). In this study, we examined negative effects on plant establishment throughout multiple demographic processes. We found that *F. natalensis* exerted opposing effects on *S*. *guineense* ssp. *afromontanum* establishment by facilitating seed deposition, seed germination, and seedling survival (Figure 1a,c,d) and by simultaneously increasing the risk of seed predation (Figure 1b).

By providing perches and fruits, a certain tree species in an open ecosystem can attract frugivores from nearby tropical forests. As a result, the seeds of these forest tree species are brought to these microsites (Begnini & Castellani, 2013; Holl, 1998). In this study, we found that seed deposition of *S*. *guineense* ssp. *afromontanum* was concentrated under *F. natalensis*, a result that is consistent with other studies that reported high densities of seeds beneath fruiting trees in savannas and abandoned pastures (Begnini & Castellani, 2013; Holl, 1998, 2002; Slocum & Horvitz, 2000). In our case, this may be a consequence of the behavior of the most common consumer of *S*. *guineense* ssp. *afromontanum* fruits, Schalow's turaco (*Tauraco schalowi*). Our previous study found that Schalow's turaco frequently visited *S*. *guineense* ssp. *afromontanum* in montane rainforests (67% of the total number of animals observed), consumed most of the fruit (63% of the total number of fruits consumed), and swallowed all of the pecked fruit (Fujita, 2014). In addition, they were frequently observed visiting fruiting *F. natalensis* in miombo woodlands (0.4 visits per hour; Fujita, 2014).

*Ficus natalensis* also had a positive effect on the germination and seedling survival of *S*. *guineense* ssp. *afromontanum*. Modifications in local abiotic conditions provide one potential explanation for the positive effects. Given that grass cover was significantly lower under *F. natalensis* compared to the treeless sites (Fujita, 2016), seedling survival can be facilitated by the reduction in fire occurrence (Hoffmann et al., 2012). This hypothesis is strongly supported by the fact that only 2% of the seedlings under *F. natalensis* were dead due to fire, which was the main cause of seedling mortality in this study. Additionally, *F. natalensis* trees reduced light penetration to the ground, creating cooler and wetter microsites that might have protected the seeds and seedlings from drought stress, especially during the dry season (Gomez, Zamora, & Boettinger, 2005; Salazar, Goldstein, Franco, & Miralles‐Wilhelm, 2012).

Contrary to seed arrival, germination, and seedling survival, *F. natalensis* may exert negative, consumer‐mediated effects on the postdispersal process before germination. Removal of *S*. *guineense* ssp. *afromontanum* seeds occurred at all four microsites, but most were removed from under *F. natalensis*. However, we do not know to what extent the removed seeds were lost to recruitment, because we did not track the final fate of the removed seeds. Although most removed seeds are eventually predated, some are able to germinate and be recruited after removal (Brewer & Rejmanek, 2009; Klinger & Rejmanek, 2009). A future study should monitor seed fate after removal and examine the consumer‐mediated effect on postdispersal stage in more detail.

Previous studies have shown that rodents generally prefer to forage under dense crowns, where the risk of predation is lower (Loayza, Loiselle, & Rios, 2011; Smit et al., 2008). In this study, however, we found significant differences in seed removal between *F. natalensis* and *B. floribunda*, while the two microsites did not differ significantly in canopy openness. Differences in food resource abundance may explain these observations. During the study period for seed removal, we found many deposited syconia, as well as seeds of montane rainforest species such as *Apodytes dimidiata* and *Diospyros whyteana* under *F. natalensis* (T. Fujita per. obs.). Iob and Vieira (2008) found that seeds in high‐density clusters suffered from higher predation than those in low‐density clusters. To understand the factors affecting postdispersal seed predation of *S*. *guineense* ssp. *afromontanum*, further study is needed.

Interestingly, when we excluded the seed arrival stage from our estimation of the transition probability, the highest values were found under *B. floribunda*, and not under *F. natalensis* (Figure 3a). This can be attributed to the higher germination and seedling survival and lower seed predation under *B. floribunda*. This result suggests that *B. floribunda* provide the most favorable microsites for the post dispersal processes of *S*. *guineense* ssp. *afromontanum* in miombo woodlands. When we included the seed arrival stage, however, the order of transition probability between *F. natalensis* and *B. floribunda* was reversed. The probability was one order of magnitude higher under *F. natalensis* than under *B. floribunda* (Figure 3b). The increased seed arrival under *F. natalensis* clearly outweighed the effect of apparent competition. Our estimate of the transition probability, which included the seed arrival stage, was consistent with natural patterns of *S*. *guineense* ssp. *afromontanum* establishment. Thus, despite contradictory effects, the net effect of *F. natalensis* on *S*. *guineense* ssp. *afromontanum* recruitment in this woodland was facilitative. The density of seed predators, such as rodents, however, is known to show interannual variation (Li & Zhang, 2007). Also, the strength of the positive effect may vary among years (Loayza et al., 2011); apparent competition for seed survival may outweigh the positive effects on seed arrival, germination, and seedling survival in some years. Thus, future studies should be conducted to monitor long‐term changes at each demographic stage.

## CONFLICT OF INTEREST

None declared.

## AUTHOR CONTRIBUTIONS

TF designed and performed the experiments. TF and CY analyzed the data. TF wrote the manuscript. TF and CY revised the manuscript.
